# Bibliometric Analysis Reveals the Progress of PM_2.5_ in Health Research, Especially in Cancer Research

**DOI:** 10.3390/ijerph20021271

**Published:** 2023-01-10

**Authors:** Yaxuan Xie, Kejian Shi, Yuncong Yuan, Meijia Gu, Shihan Zhang, Kai Wang, Liangying Fu, Chao Shen, Zhanpeng Yuan

**Affiliations:** 1 School of Public Health, Wuhan University, Wuhan 430000, China; 2 College of Life Sciences, Wuhan University, Wuhan 430000, China; 3 Key Laboratory of Combinatorial Biosynthesis and Drug Discovery, Ministry of Education, School of Pharmaceutical Sciences, Wuhan University, Wuhan 430000, China; 4 Hubei Provincial Key Laboratory of Applied Toxicology, D1 Safety Assessment Center, Bio-City Innovation Park, Wuhan 430000, China

**Keywords:** PM_2.5_, cancer, bibliometrics, Web of Science, VOSviewer, CiteSpace, co-occurrence analysis

## Abstract

PM_2.5_ has an aerodynamic diameter of less than or equal to 2.5 microns due to its inherent physical and chemical properties so that it can enter the alveoli through the respiratory tract for blood gas exchange. Numerous studies have shown that PM_2.5_ is a serious air pollutant that poses a wide range of health risks, especially for cancer. Bibliometric methods were employed to have comprehensively analyzed the research of PM_2.5_ in cancer for about a decade in Web of Science to identify hotspots and trends using VOSviewer, CiteSpace, and R. The field has undergone overall growth in the past decade. As research on PM_2.5_ in health deepens, cancer related to it expanded beyond the respiratory system to the digestive system, urinary system, female gonadal axis, breast cancer and other cancers. Another observation is that research on PM_2.5_ in cancer has progressed in the mechanisms of deterioration, such as the role of matrix metalloproteinases in cancer. In addition, research on the risks of PM_2.5_ in combination with polycyclic aromatic hydrocarbons and heavy metals has also emerged. Results showed that there are relatively more studies on PM_2.5_ in high-latitude countries, which may be due to different national conditions, such as climate and coal combustion. Our research has combed through the progress of PM_2.5_ in cancer research and provided a supplement for developing pollution prevention ideas with different national conditions in this field.

## 1. Introduction

Air pollution-caused health problems, primarily in the form of PM_2.5_, have been a growing public health concern, and are considered to be a consequence of social economic development driven by urbanization and industrialization. Hence, air pollution greatly affects human health and social development [1]. PM_2.5_ has an aerodynamic diameter of less than or equal to 2.5 microns. Due to its inherent physical and chemical properties, it can enter the alveoli through the respiratory tract for blood gas exchange [2]. PM_2.5_ poses a significant threat to health. Murray et al. reported that PM_2.5_ ranked sixth among level four risk factors for death. The particulate pollution burden of Global Burden of Disease (GBD) 2019 was 44.6% higher than that of GBD 2017, showing a significant upward trend [3]. Therefore, scientists and national leaders should pay attention to the study of PM_2.5_.

Exposure to PM_2.5_ is closely related to the increased mortality of lung and cardiovascular diseases (such as chronic obstructive pulmonary disease, lung cancer, heart failure and hypertension) in epidemiological studies [4,5,6,7]. The research on its mechanism has mainly focused on oxidative stress, inflammatory response mechanism, the role of matrix metalloproteinases in its deterioration, etc. [8].

With the systematization and maturation of research in this field, exposure to PM_2.5_ could also worsen central nervous system diseases, such as depression and Alzheimer’s disease [9,10], and induce immune system tumors, such as non-Hodgkin’s lymphoma [11], which also has a corresponding burden on blood diseases, such as inducing and aggravating blood cancer (leukemia) [12].

Bibliometrics first appeared in the early 20th century. It formed an independent discipline and was widely used in literature analysis [13,14]. Bibliometric analysis provides a quantitative method for reviewing and investigating existing literature in specific fields [15]. In the analysis process, it can obtain detailed information such as authors, keywords, journals, countries, institutions, and references. Thus, the development of an area can be obtained through bibliometric analysis [16]. Co-citation is also often used in bibliometric analysis. If one or more other articles cited the same two articles at the same time, they were defined as relationships (visual analytics). Some researchers emphasized that the visualization method of co-citation analysis in bibliometrics was helpful for data interpretation, making the results more comprehensive [17].

Currently, most research is conducted in subdisciplines and does not have an overall intuitive visual correlation analysis. Bibliometrics connects various subjects and can accurately locate keywords and present them to everyone in a visual way, providing connections and intuitive effects that ordinary reviews cannot achieve. In this study, we conducted a bibliometric analysis of articles on the effects of PM_2.5_ on cancer from 2012 to 2022 in Web of Science. We tracked research hotspots and issues in the field, hoping to provide analysis and predictions for researchers studying the field.

## 2. Materials and Methods

### 2.1. Data Source and Search Strategy

We searched the publications in the Web of Science Core Collection (WoSCC) database using two keywords: “PM_2.5_” and “Cancer” from 2012 to 2022. Keyword searches included paper titles, abstracts and keywords. A total of 2417 articles were obtained. There were 693 articles after screening by the research areas oncology/public environmental occupational health/medicine general internal. We conducted a manual review to examine the content of each article (including paper title and abstract) to eliminate repeated and irrelevant publications to ensure that the selected articles were about PM_2.5_ and cancer. Finally, 297 articles were retained for analysis (Table 1).

### 2.2. Bibliometric Analysis and Visualization

The bibliographic information of the selected publications was converted and analyzed automatically by the Bibliometrix package in R 4.1.1. We assessed publication quality by author based upon metrics that included the number of publications, citations in the research area, publication h-index value, and m-index value [18].

Networks and visualizations were constructed using VOSviewer (Version 1.6.18): co-authorship analysis of countries/institutions/authors, co-citation analysis of journals/references, citation analysis of documents, and co-occurrence analysis of keywords. We used the default parameters.

CiteSpace (5.8.R3) was used to analyze basic statistical indicators and time zone effects of keywords. In the CiteSpace 5.8.R3 interface, the time span was set to “2012–2022” (slice length = 1), threshold 7.

## 3. Results

### 3.1. The Trend in Global Publications

**Figure 1:**
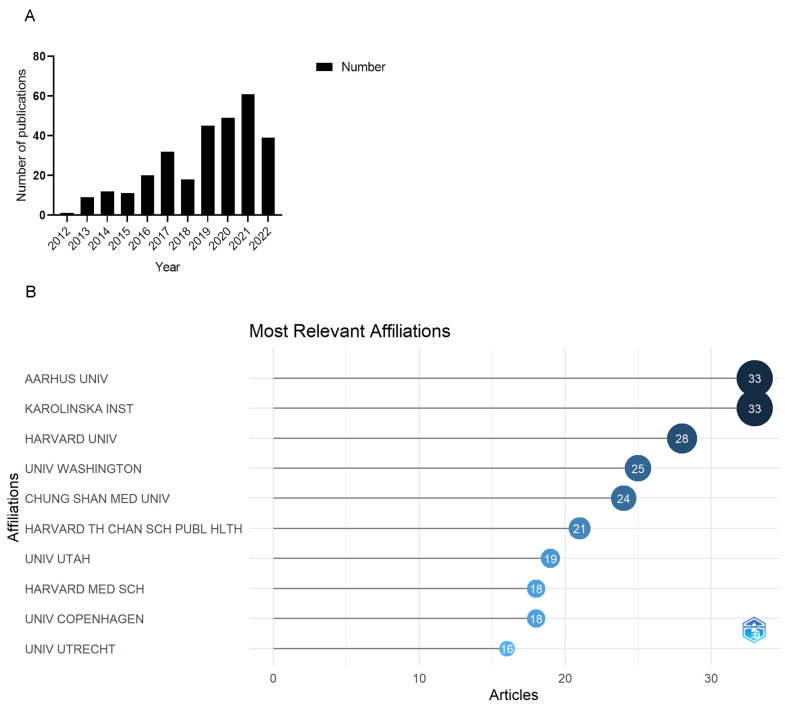
(**A**) Publication outputs and time trend. (**B**) Top ten affiliations with the largest number of publications.

There were 302 articles from 2012 to 2022 related to PM_2.5_ and cancer complying with standards retrieved from WosCC in Figure 1A. From 1 article in 2012 to 61 articles in 2021, global publications in the field showed a growth trend. This meant that the international community had paid extensive attention to issues related to PM_2.5_ and cancer. The number of articles in 2022 was lower than that in 2021, probably because we only collected articles published before 30 September 2022.

### 3.2. Distribution of Institutions

The institution distribution of articles was analyzed by R (Figure 1B). A total of 563 institutions contributed to publications in this field. Aarhus University and Karolinska Institute have published the most articles (33), followed by Harvard University (28), University of Washington (25), Chung Shan Medical University (24), and Harvard T.H. Chan School of Public Health (21).

### 3.3. Analysis of Journals and Research Areas

**Figure 2:**
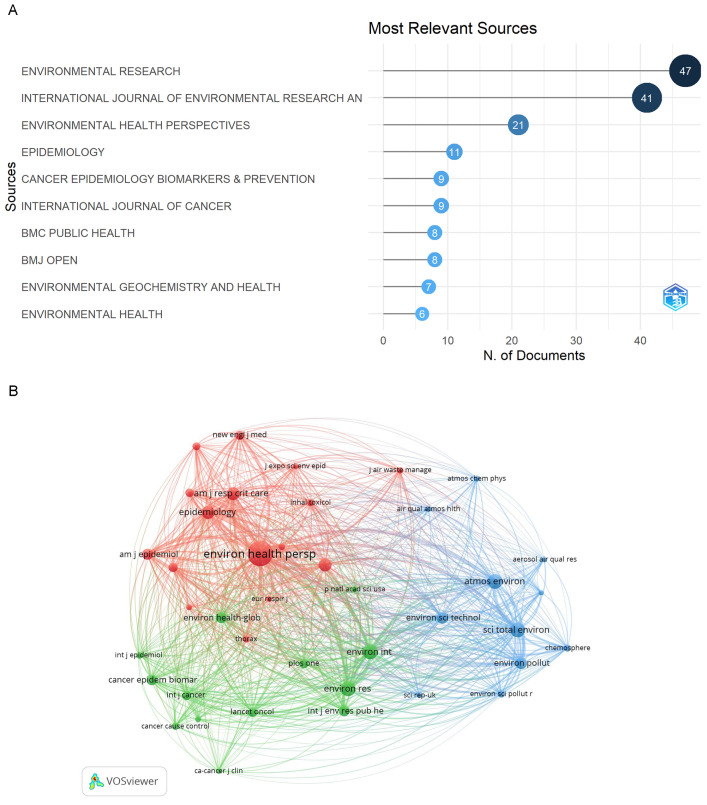
Analysis of journals. (**A**) Top ten journals for number of publications. (**B**) Network visualization of top 40 co-cited journals, and different colors represented different clusters.

In sum, 297 articles were published in 90 journals. Figure 2A shows the 10 most popular journals for PM_2.5_ and cancer analyzed by R. These journals were about public health, environment and cancer research.

We also analyzed co-cited journals in this field using VOSviewer. A total of 2732 journals were cited and Figure 2B shows the top 40 co-cited journals. *Environmental Health Perspectives* had the greatest number of citations (1022 citations), followed by *Environmental Research* (361 citations), *Atmospheric Environment* (353 citations), *Science of the Total Environment* (348 citations), and *Environment International* (340 citations). These cited journals were grouped into three categories (Figure 2B). The red cluster represents environmental epidemiology, the green cluster oncology and health, and the blue cluster environmental pollution and particulate matter.

### 3.4. Analysis of Authors and Publications

**Figure 3:**
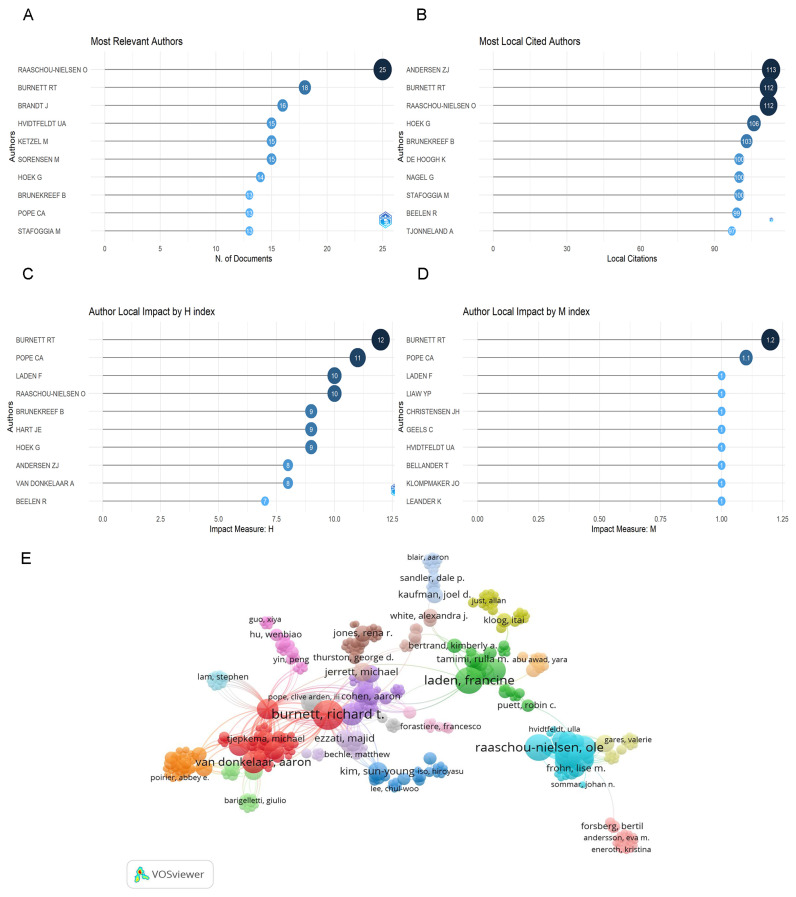
Analysis of authors. (**A**) Number of publications from different authors. (**B**) Total citations in the research filed from different authors. (**C**) H-index of publications from different authors. (**D**) M-index of publications from different authors. The above are listed in the top ten. (**E**) Network visualization between the most connected authors.

Of all the authors of 297 articles, Raaschou-Nielsen was the most productive, with 25 articles (8.4% of all articles), followed by Burnett (18, 6%), Brandt j. (16, 5.4%) (Figure 3A).

The most cited author was Andersen zj. (113 citations), followed by Burnett and Raaschou-Nielsen (112), Hoek j. (106) (Figure 3B).

Articles of Burnett had the highest h-index, followed by Pope ca., Laden f. and Raaschou-Nielsen in Figure 3C. The m-index of publications of Burnett also ranked the first, followed by Pope ca. and Laden f. in Figure 3D.

We analyzed all 1619 authors, Figure 3E showed links between them. Figure 3E showed the most oversized connected items consisted of 428 items. The five most closely related authors were Burnett (150 total link strength), Raaschou-Nielsen (117), Brandt j. (107), Geels c. (106) and Van donkelaar a. (106) (Figure 3E).

### 3.5. Co-Citation Analysis

**Figure 4:**
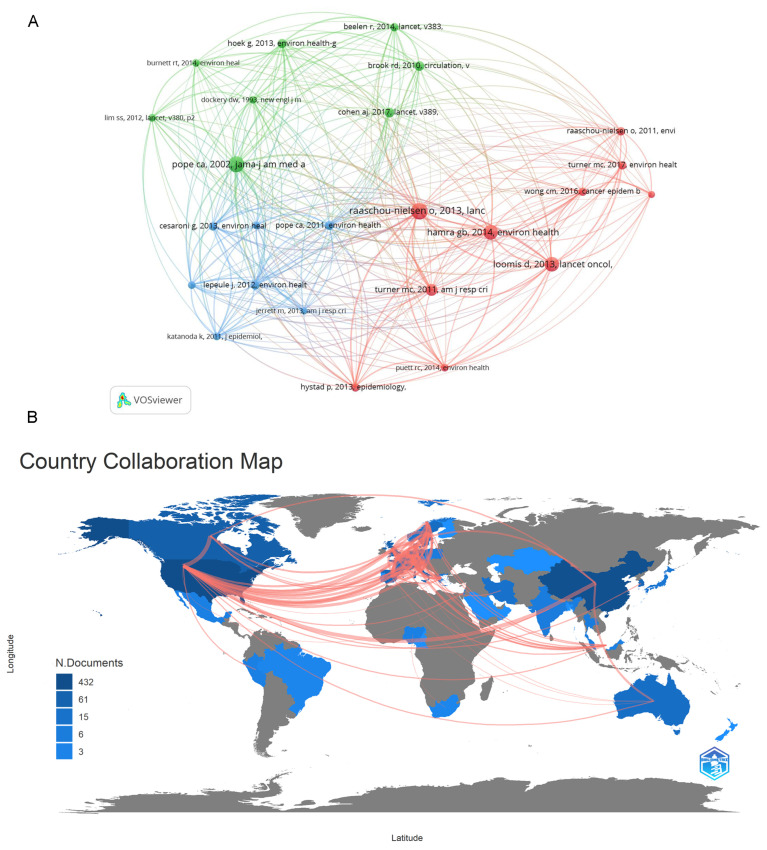
(**A**) Network visualization relations of articles cited 20 times or more. (**B**) Country collaboration map of the world. The red line represented the contact and cooperation between countries. The left corner of (**B**) showed the statistics of each author’s country.

There were 25 articles co-cited 20 times or more. “Air pollution and lung cancer incidence in 17 European cohorts: prospective analysis from the European Study of Cohorts for Air Pollution Effects (ESCAPE)” Ole Raaschou-Nielsen had 61 citations in Lancet Oncol, “Lung cancer, cardiopulmonary mortality, and long-term exposure to fine particulate air pollution” C Arden Pope 3rd had 57 citations in JAMA, and “ Outdoor particulate matter exposure and lung cancer: a systematic review and meta-analysis” Ghassan B Hamra had 51 citations in Environmental Health Perspect (Figure 4A).

### 3.6. Country Collaboration Analysis

We have made a world map of collaboration among countries and found that most of the current researches in this field came from developed countries, a few from developing countries in Figure 4B. It can be seen that the darker the color is, the more authors there are in this country. China pays more attention to lung cancer and its mechanism research, while the United States covers a wide range of research areas in PM_2.5_ and cancer research. There have been 19 cooperations between China and the United States, and China and the United States mainly cooperate in research on lung cancer and brain tumor. There have been 18 cooperations between the United States and the United Kingdom and 13 cooperations between United States and Demark mainly in lung cancer. The United States and Mexico cooperate in research on breast cancer. Sweden, Denmark, the United Kingdom, the Netherlands, Germany and other European countries have cooperated in the research of lung cancer, breast cancer, bladder cancer, stomach cancer and brain tumor (total 81 cooperations).

### 3.7. Co-Occurrence Analysis of Keywords

**Figure 5:**
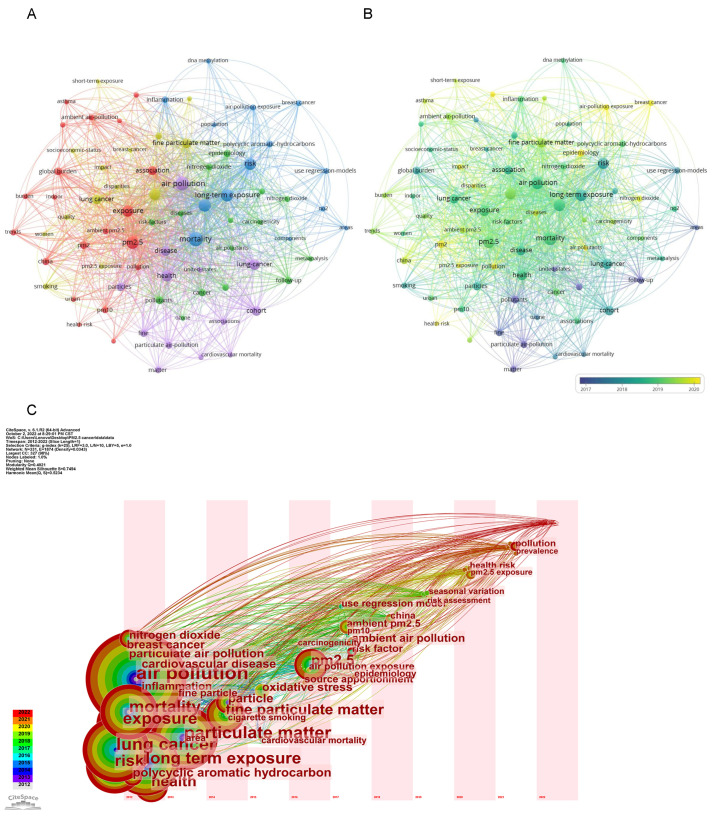
Co-occurrence analysis of keywords. (**A**) Network visualization of keywords of studies. (**B**) Distribution of keywords according to average publication year (blue: earlier, yellow: later). (**C**) The visualization of timezone of keywords from 2012 to 2022. We can see the publication time at the bottom of the figure, the line between each keyword represented their connection.

We analyzed 50 keywords that occurred more than 10 times in Figure 5A. Overlay visualization indicated the average publication year of the identified keywords (Figure 5B). Timezone visualization could understand research hotspots horizontally and vertically, which could more intuitively see the changes in research hotspots with the time axis and the duration of hotspots (Figure 5C).

**Figure 6:**
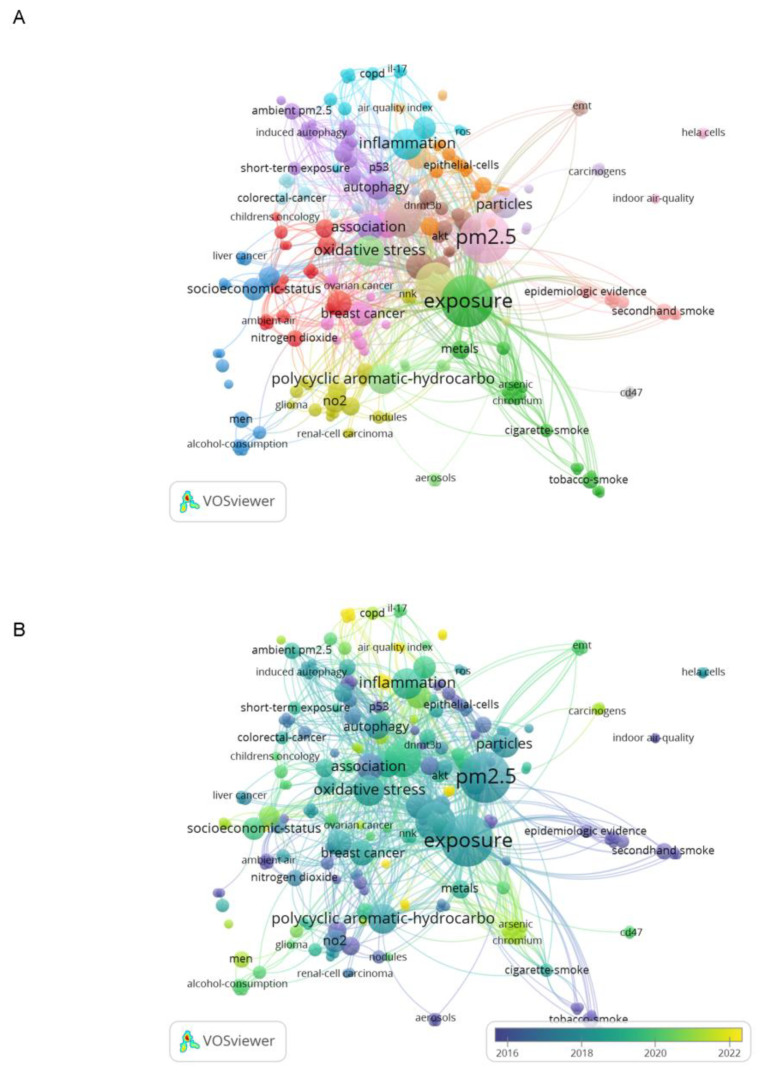
Co-occurrence analysis of keywords of fine-extracted 94 articles. (**A**) Network visualization of keywords of studies. (**B**) Distribution of keywords according to average publication year (blue: earlier, yellow: later).

We fine-extracted 94 articles about PM_2.5_ and cancer except lung cancer, and their keywords were analyzed for co-occurrence analysis. Because there are many studies on lung cancer about PM_2.5_, and the circle in the figure is large, which will cover the circle of other cancer studies. We want to see the status of other cancers in PM_2.5_ research, we screened these articles manually. One color represented a kind of clustering in Figure 6A, and for example, light blue represented the general content of mechanism research like inflammation and IL-17 in cancer research. Figure 6B is the overlay visualization, the color from dark to light represents the year from older to present. We can see that as PM_2.5_ in cancer research is deepening, the content was becoming more extensive and systematic, from respiratory cancers to digestive and reproductive system and more.

## 4. Discussion

In this study, we characterized the current landscape of PM_2.5_ and health problems like cancer research by bibliometric analysis and network visualizations. We analyzed the contributions of countries, institutions, journals and authors to the global concerns. We also viewed focuses in the past and predicted hot topics that would be of continued research interest in the future. The number of articles published in this field has been growing steadily since 2012, and an outbreak in 2021. According our results, Aarhus University and Karolinska Institute is the currently the world leader in PM_2.5_ and cancer research. The results showed that major contribution in this field were from Europe and the United States with relatively developed and mature works. With the global economic integration, some developing countries, such as China, have also begun to pay attention to air pollution, and accelerated the research in this field [19,20,21,22].

### 4.1. Journals Contribution about PM_2.5_ and Cancer

The top ten journals in PM_2.5_ and cancer were about environment, epidemiology, and cancer research. The visualization of the top 40 cited journals showed that the journals were mainly clustered into three categories. Red cluster was about environmental epidemiology, green cluster was about cancer and medicine research, and blue cluster was about atmospheric particulate pollutants. These results meant that PM_2.5_ had been widely studied in environmental science, chemical science, and biomedicine, which also showed a trend of multi-field research in the future.

### 4.2. Authors Contribution about PM_2.5_ and Cancer

Raaschou-Nielsen was the most prolific author in PM_2.5_ and the cancer field with 25 publications, followed by Burnett. The h-index and m-index of Burnett were higher than Raaschou-Nielsen, but all of them were in the top five. This showed that their articles are of high quality and broad influence. Raaschou-Nielsen, O. mainly concentrated on risk assessment of air pollutants and human health, especially lung cancer [21]. Burnett mainly focused on the impact of PM_2.5_ in the air on human disease burden [11,23,24].

### 4.3. Cited Articles and Country Collaboration about PM_2.5_ and Cancer

Co-citation analysis showed the top three cited articles mainly demonstrated the relationship between PM_2.5_ and lung cancer, and the burden of PM_2.5_ on the cardiopulmonary system [25,26,27]. A country collaboration map told us the research on PM_2.5_ and cancer started from developed countries and then flowed to developing countries. This might be because people in developed countries tend to spend more on health care after they reach a level of material and living prosperity, while developing countries are in a rapid development stage and often neglect attention to health care. We also found most countries with strong researches are in high-latitude regions. This might because these regions need to use coal and other substances for heating in winter, and if coal and other materials are not burned sufficiently, particles are produced. This is more likely to exposure to particulates in the air than countries with low latitude. Most of the studies on PM_2.5_ and lung cancer came from China. Some of these were mechanism studies. A majority of gynecological cancers such as breast cancer (such as ovarian cancer) came from the United States, and the United States had a wide range of PM_2.5_ and cancer research. Taiwan and China, tended to study chronic or long-term exposure, with research on oral cancer and nasopharyngeal cancer. In addition to lung cancer, Danish research on PM_2.5_ and cancer mainly focused on leukemia (blood cancer) and non-Hodgkin’s lymphoma. Sweden, Denmark, the Netherlands, the United Kingdom and another three countries in Europe mainly carried out large cohort studies, focusing on lung cancer, liver cancer, breast cancer, bladder cancer and brain tumors. Peru had a study on the impact of early exposure to PM_2.5_ on neonatal tumors in pregnancy outcomes.

### 4.4. Keyword Co-Occurrence Analysis for PM_2.5_ and Cancer

We used Figure 5 to show the co-occurrence analysis of keywords. We can clearly see that the most prominent keywords in this field were air pollution, PM_2.5_, long-term exposure, lung cancer, and mortality. The keywords were clustered into five categories. The red cluster was mainly for exposure factors, yellow mainly for short-term acute exposure and its results, blue mainly for outcomes of long-term exposure and inflammation mechanisms, green mainly for other pollutants combined with PM_2.5_, and purple cluster health burden results. Our results suggested that research trends were moving from long-term exposure to short-term exposure, from lung cancer to cardiovascular and cerebrovascular diseases to women ‘s cancers, such as breast cancer. From the time zone chart, the keywords with large circles are those that have continued to heat up since 2012. Horizontally, you can see PM_2.5_ and the evolutionary history of cancer research. The research hotspots have been subdivided from extensive air pollution to particulate matter PM_2.5_, the outcomes have been gradually subdivided from mortality to various systemic diseases such as lung cancer and cardiovascular disease, and the mechanisms have been gradually subdivided from inflammatory manifestations to oxidative stress and IL-17 and more [8], and researchers have begun to consider seasonal changes [8,28]. Adverse health effects of PM_2.5_ were further discerned from some other air pollutants, such as polycyclic aromatic hydrocarbons (PAHs) and heavy metals. China as a developing country has also gradually appeared on the keyword time zone map, indicating that developing countries were increasingly focusing on environmental friendliness and sustainable development. In addition to more in-depth studies on diseases caused by PM_2.5_, the size effect and mechanisms were also studied. Some studies had shown that the smaller the particles, the easier toxic substances are adsorbed, thus entering the human body [29]. In terms of binding with other pollutants, polycyclic aromatic hydrocarbons were the most common, followed by heavy metals. The combination of PAHs and PM_2.5_ was mainly from vehicle emissions, industrial activities, and fuel burning [30,31]. This also indicated that the synergistic effects of PM_2.5_ combining with other pollutants were beginning to be explored based on their characteristics. In conclusion, research on PM_2.5_ and cancer is becoming more detailed and systematic, and social and economic factors are taken into account [22].

### 4.5. Keyword Co-Occurrence Analysis for PM_2.5_ and Cancer (Except Lung Cancer)

We also manually reviewed and examined 94 articles about PM_2.5_ and cancer closely, and conducted cluster analysis on their keywords to track hotspot changes. As shown in Figure 6A, research hotspots of lung cancers and cardiovascular diseases caused by PM_2.5_ show a trend of subdivision by sex, age and system in recent years. Blue clustering showed that men were more likely to suffer from digestive system cancers after exposure to PM_2.5_, such as liver cancer and colorectal cancer. Pink clustering showed that women were more likely to have female systemic cancers, such as breast cancer and ovarian cancer [32,33,34]. From Figure 6B of time visualization, in recent years, some studies have begun to explore the association between PM_2.5_ and urinary system cancers, such as bladder cancer and renal cancer, as well as the impact on the immune system, such as non-Hodgkin’s lymphoma, and the association with leukemia [12,35]. The study of PM_2.5_ causing brain and nervous system lesions had always existed, and studies had shown that it is related to the adverse outcomes of Alzheimer’s disease [36].

In view of the extensive and severe harm of PM_2.5_ to human health, countries around the world have taken a series of measures to prevent air pollution and reduce particulate matter content in pollutants. However, the PM_2.5_ content in the air of some countries is still above the limit. This is not only related to the health and economic development of one country’s population, because the atmosphere is flowing and will cause a global impact with the airflow. Therefore, the study of PM_2.5_ on health, especially cancer, a disease of this malignant outcome, is necessary. Countries should intervene in the health damage caused by PM_2.5_ in combination with their national conditions and proceed with long-term studies on health damage caused by PM_2.5_.

## 5. Conclusions

Research on PM_2.5_ and cancer is becoming more detailed and systematic, showing a trend of subdivision by sex, age and system in recent years. Countries should conduct long-term research on the health damage caused by PM_2.5_ and propose preventive measures according to their national conditions.

## Figures and Tables

**Table 1:** Data source and search strategy.

Category	Specific Standard Requirements
Research database	Web of Science core collection
Citation indexes	SCI
Searching period	30 September 2012 to 30 September 2022
Searching keywords	(“PM_2.5_”) AND (“Cancer”)
Subject categories	Oncology/Public Environmental Occupational Health/Medicine General Internal
Document types	“Articles”, “Reviews”, “early access”, “proceedings paper”
Data extraction	Export with full records and cited references in plain text format
Sample size	297

## Data Availability

Not applicable.
